# Patient’s and Consultant’s Views and Perceptions on Switching from an Originator Biologic to Biosimilar Medication: A Qualitative Study

**DOI:** 10.3390/pharmacy12020065

**Published:** 2024-04-07

**Authors:** D. C. Rosembert, M. J. Twigg, D. J. Wright

**Affiliations:** 1 Pharmacy Department, Cambridge University Hospitals NHS Foundation Trust, Cambridge CB2 0QQ, UK; 2 School of Pharmacy, University of East Anglia, Norwich NR4 7TJ, UK; m.twigg@uea.ac.uk; 3 Research and Innovation Team, NHS Norfolk and Waveney ICB, Norwich NR4 7TJ, UK; 4 School of Healthcare, University of Leicester, Leicester LE1 7RH, UK; d.j.wright@leicester.ac.uk

**Keywords:** biologics, biosimilar, switching, autoimmune disease, monoclonal antibodies, adverse drug reactions, education, pharmacist

## Abstract

The aim of this study was to describe the opinions of patients undergoing treatment with originator biologics and medical consultants managing their conditions and identify the barriers and enablers to transitioning from originator biologics to equivalent biosimilars. This study was undertaken prior to biosimilar switching at a large teaching hospital in the United Kingdom. Five gastroenterology, rheumatology, and dermatology consultants were interviewed. Two focus groups were conducted with patients prescribed infliximab (n = 2) and etanercept originators (n = 7). Four main themes emerged, as follows: (1) ‘Benefit to the NHS’; (2) ‘Evidence for efficacy and safety’; (3) ‘Team roles’; and (4) ‘Effective communication during switching’, with sub-themes such as (4a) ‘What patients want to know’ and (4b) ‘How it should be communicated’. Recognition of the ability to save NHS money was an enabler for both patients and consultants, with patients wanting to be reassured that the money saved would be used to benefit other patients. Consultants did not always believe that biosimilars had similar efficacy as the originators or that the manufacturing standards were the same. Effective interventions to address these concerns are required. Offering patients the opportunity to revert back to their originator if necessary was seen as an enabler, as was the provision of readily available mechanisms for reporting suspected adverse events resulting from switching. The role of pharmacy in the process of switching from originator biologics to biosimilars can range from educating consultants regarding the safety and efficacy of biosimilars, explaining the rationale for switching patients, and providing a route for reporting adverse events.

## 1. Introduction

Biologics are a diverse group of medicines that include vaccines, growth factors, immune modulators, monoclonal antibodies, and products derived from human blood and plasma. They are distinguished from other medicines as they are complex molecules, i.e., proteins purified from living culture systems or blood, whereas small-molecule medicines are made synthetically [1].

Biologics work by mimicking and inhibiting different proteins within the immune system that cause inflammation, where there is overproduction of the natural protein or enhanced sensitivity to it [2,3,4]. Due to the complexity of these molecules and the cost of production, the market price is frequently relatively high. Consequently, they have been traditionally prescribed or allowed to be used only when other options have already been tried and failed. Due to their cost, biologics are rarely first-line therapy and more frequently third- or fourth-line.

Biosimilar medicines contained the same active substance as an approved and licenced biologic and were first introduced in 2006. Competitor companies can only launch biosimilars once the protection provided by the original marketing authorisation has expired [5]. They are usually significantly cheaper to acquire and, therefore, provide an opportunity to treat more patients earlier on in the prescribing pathway.

Biosimilars are, however, not considered exactly the same as their originator molecule but have met regulatory requirements by demonstrating similar efficacy and safety in at least one clinical trial representing a licenced indication of the originator molecule [6]. As they are not truly identical in the same way that generic small-molecule-based medicines are compared to the original product with the marketing licence, greater concerns exist regarding the transfer from biologics to biosimilars than those that occur when moving from a branded simple molecule to a generic one. Evidence acquired over a number of years of clinical use shows that biosimilars approved through the EMA (European Medicines Authority) can be used safely and effectively in all their approved indications when compared to originator medicines [7,8].

The United Kingdom (UK) health system budget for medicines on items dispensed in hospitals in England, of which a proportion includes biologics and biosimilar medicines, has increased significantly from GBP 6.63 billion in 2017 to GBP 9.45 billion in 2023, which is a 42.5% increase. Without the addition of biosimilars to the market, the increase in spending would have been considerably higher [9]. By 2021, it was estimated that the UK system could save at least GBP 300 million per year if the best-value biosimilars were used instead of originator molecules [5]. These savings, however, have not been fully realised, with researchers reporting some barriers to biological switching. Changing prescriber behaviour to more routinely use biosimilars has proven to be more difficult than anticipated. A recent survey conducted with paediatric rheumatologists showed that gaps in prescriber knowledge and insufficient familiarity remain valid challenges for the adoption of biosimilars, and this has been seen when extrapolating to other clinical areas with little experience in using biosimilar medicines [10]. Many studies have since provided reassurance that switching from reference biologics to biosimilars is effective and safe [11]. A survey showed that the most significant barrier to overcome for biosimilar adoption was prescriber education about evidence from switching studies, with the lowest being the requirement for therapeutic drug monitoring for switched patients [12]. A systematic review reported that patients often felt inadequately informed about biosimilars, leading to doubts about their clinical effects and regulatory approval pathway, potentially influencing their attitudes negatively [13]. Moreover, study findings recognise the broader educational gap by highlighting that concerns may arise not only from biosimilars themselves but also from their originators and variations among batches [14].

Consultants’ communication strategies regarding biosimilar medicines can greatly influence patient experiences, particularly if their attitude towards the intervention is negative. Research has extensively documented the occurrence of the nocebo effect, wherein patients may experience symptoms based on negative expectations regarding treatment, both in clinical trial settings and routine care contexts. This phenomenon can significantly impact clinical outcomes and lead to the discontinuation of treatment. Therefore, for a successful transition to biosimilars, it is crucial that patients are thoroughly informed and comprehend the background and rationale behind the switch. They should also have the opportunity to voice any concerns they may have about initiating or transitioning to biosimilars. Effective communication from consultants can help alleviate fears and misconceptions, thereby reducing the likelihood of the nocebo effect influencing patient outcomes. By addressing patient concerns and providing comprehensive information, consultants can enhance patient confidence in the transition process and promote better adherence to biosimilar therapies, ultimately contributing to improved healthcare outcomes [15,16,17].

Real-world data and publications of position statements from the Royal Colleges, British Society of Gastroenterology (BSG), British Society of Rheumatology (BSR), and British Association of Dermatology (BAD) also provide patients and consultants with reassurance [18,19].

Examples of biologics that have significant financial consequences for the health system and potential for large savings with greater use of biosimilars in the treatment of autoimmune diseases are infliximab and etanercept through the expiry of their patents in 2013 and 2016, respectively. Cambridge University Hospitals NHS Foundation Trust, a large teaching hospital in the UK, provided an opportunity to understand local perspectives to inform the introduction of the switching from biologic originators to biosimilars process. We, therefore, carried out a qualitative study to explore the views and perceptions of patients receiving treatment with biologics and the consultants managing their disease to identify barriers and enablers to the switching process. This study provided a unique opportunity to approach patients with autoimmune diseases, including inflammatory bowel diseases (Crohn’s disease and ulcerative colitis), rheumatoid arthritis, psoriatic arthritis, and psoriasis, as well as their consultants, prior to them using biosimilar medicines.

## 2. Materials and Methods

### 2.1. Study Overview

This study used a pragmatic qualitative approach to answer the research question. Focus groups were conducted with patients who were prescribed originator molecules and had not been switched to biosimilar medicine. Individual interviews were conducted with medical consultants who had only prescribed originator molecules. Focus groups were chosen for patients because group dynamics and collective views on a topic were desired. Structured interviews with open-ended, predetermined questions were chosen for consultants because, at the time, it was a topic that required in-depth exploration, and each specialist consultant might have had different concerns to explore [20]. The primary researcher, supervised by the co-authors, is a female, registered pharmacist who had completed the research methods module as part of a Master of Science degree at the University of East Anglia prior to undertaking this study. The co-authors are both academic pharmacists.

### 2.2. Relationship with Participants and Patient Recruitment

There was no personal relationship established with the participants prior to study commencement. The primary researcher, who was also the lead pharmacist for biologics, was the only member of the research team who could access patient details to request expressions of interest to join the study.

Patients prescribed infliximab and etanercept were identified from the Trust’s electronic prescribing system (Epic 2014) and the Trust’s homecare patient database (Microsoft Office MS Access 2016). A cohort of 40 patients was selected at random from the overall list using the RAND formula in Microsoft Office MS Excel 2016. From the list of 40 patients, the first 10 participants were prescribed infliximab originator medicine, and 10 participants were prescribed etanercept originator medicine. They were selected, starting from the top of the list, to receive an invitation to the focus groups. The patients were contacted via post to express interest in participating in a focus group. Patients were sent an expression of interest (EOI) letter and consent form, which included the logo of the University of East Anglia and the Cambridge University Hospitals, along with a pre-paid stamped envelope to return the EOI letter. The reasons and interest in the research topic were communicated to the participants within the participant information leaflet (PIL) and covering letter.

Inclusion criteria: The patients were established either on infliximab or etanercept originator for a minimum of 6 months, as the patients would have had follow-up appointments to check disease activity and for response to the medicine as per NICE guidelines [21]. Consultants could be reassured that the patient is established on the treatment and has had a response to the medication; therefore, they are candidates to switch to a biosimilar medicine. These patients would also have had sufficient experience being on the medicine to become familiar with their treatment. The patients had to be diagnosed with either:

- immune medicated inflammatory diseases (IMIDs) such as rheumatoid arthritis (RA), ankylosing spondylitis (AS), and psoriatic arthritis (PsA)
- conditions affecting the skin, e.g., psoriasis (PS)
- inflammatory bowel disease (IBD), including Crohn’s disease (CD) and ulcerative colitis (UC) >18 years of age

There was an 80% response rate from participants returning their consent forms with the initial round of invitations. However, the majority could not attend on the designated day of the focus group but offered to participate at another time. Two participants from the infliximab group confirmed attendance, and the decision was made to go ahead with the focus group as a pilot as it would be highly informative to obtain views from these patients as well as an opportunity to test the length and flow of the questions where appropriate. A second round of invitations was sent out to a further 40 patients selected at random from the etanercept originator list for the focus group; seven participants attended the session.

### 2.3. Consultant Recruitment

Expressions of interest to participate in this study were sent to the medical consultants employed at Cambridge University Hospitals who prescribe biologics and work in specialist areas of dermatology, rheumatology, and gastroenterology. The expression of interest was sent via email, and then a convenient date and time for the 45 min interview were arranged. Interviews were conducted in November 2016 by the primary researcher and recorded using a digital voice recorder (Olympus W5-550M) and transcribed verbatim.

### 2.4. The Focus Groups and Consultant Interviews

The focus groups took place on two separate occasions, in September 2016 and October 2016, at the Cambridge University Hospitals campus. The focus groups were conducted in a seminar room on the hospital site, and the primary researcher and facilitator were present. The consultant interviews were conducted in the consultant’s office, where only the primary researcher was present as a non-participant.

The focus group structure included a brief presentation to provide participants with foundational knowledge before discussing questions. This presentation covered comparisons between biologics and biosimilars, including the manufacturing process, mechanism of action, devices, cost differences, and route of administration. Its purpose was to minimise bias and ensure all participants had a uniform baseline of knowledge from healthcare professionals rather than the media. The presentation strictly presented factual information without including opinions or views, focusing solely on basic facts about the two types of medicines.

The focus groups were recorded using a digital voice recorder (Olympus W5-550M) and transcribed verbatim.

The questions asked in the focus group and consultant interviews were designed to allow multiple perspectives of participants’ perceptions and experiences to emerge during the interview. Specific content, such as how switching to a biosimilar might or might not impact adherence, preferred methods to communicate the information regarding the change to a biosimilar to patients, what information is deemed to be required, the timing of the switch, and which health care professionals should be involved, was discussed.

Topic guides (Appendix A and Appendix B) were developed, detailing the structure, prompts, and questions to be asked during the interviews and focus groups. These were not shared with the participants.

### 2.5. Data Collection

The infliximab originator focus group was the pilot in order to test the topic guide; following this, no changes were identified as being required within the process. No repeat interviews were conducted. The interviews were conducted using a digital voice recorder (Olympus W5-550M) and transcribed verbatim.

The recording from the focus groups and one-to-one interviews was transcribed and anonymised so that participants would not be identified; each participant was assigned a number. The transcripts were not returned to participants for comments or corrections. The co-researchers also contributed by reading the transcripts and reviewing the themes presented by the primary researcher.

### 2.6. Data Analysis

A thematic analysis was conducted by the primary researcher using Nvivo Pro 11^®^. The co-researchers, who are both academic pharmacists, also supported the analysis by reading the transcripts and the themes presented. The final thematic analysis was checked by both co-researchers. The six phases of thematic analysis described by Braun and Clarke, which include familiarising oneself with data (comments), generating initial codes, searching for themes, reviewing themes, defining and naming themes, and producing the report, were followed [22,23].

The documented process included six phases. Firstly, the researcher transcribed the recording verbatim, including verbal and non-verbal responses. Once transcribed, the data were read repeatedly, and initial ideas were noted to ensure these data were familiar. Secondly, the transcripts were uploaded to Nvivo Pro 11^®,^ and initial codes were generated. Thirdly, transcripts were coded inductively, with these codes gradually combined into potential categories and, subsequently, themes within the data across the focus groups and consultants’ interviews. The fourth stage of the process involved reviewing the themes in relation to the coded extracts and the entire data set repeatedly. The fifth and sixth phases allowed the generation of clear definitions for each theme, as the specifics of each theme were defined and named to create the results reported. The analysis was conducted immediately following the completion of interviews and focus groups.

### 2.7. Governance

This study required ethical approval because there was direct involvement with patients; it was confirmed by the Health Research Authority (IRAS ID: 199526) and registered with the University of East Anglia and Cambridge University Hospitals Research and Development departments.

## 3. Results

A total of nine patients and five consultants participated in the study. Table 1 describes the demographics and diagnoses of the patient participants. The infliximab originator focus group includes two participants. The etanercept originator focus group included seven patient participants, and four partners were also present for support. The partners remained in the room, but they did not participate in the discussion.

The characteristics of five consultant participants in face-to-face interviews included consultants from gastroenterology, dermatology, and rheumatology specialities. None of the consultants had prescribed a biosimilar medicine at the time of this study.

The transcripts were uploaded to Nvivo Pro 11^®^, where 61 codes were retrieved, and initial quotes and codes were identified from emerging themes within the data across the focus groups and consultant interviews.

The four themes are (1) Benefits to the NHS; (2) Evidence for efficacy and safety; (3) Team roles; (4) Effective communication during switching—(4a) What patients want to know and (4b) How it should be communicated.

(1) Benefit to the NHS

There was consensus regarding the benefits of moving from biologics to biosimilars. The money freed from the transfer could be used elsewhere within the NHS or to enable more patients to access the treatment.

*‘cost, to my mind, actually also directly translates into access to the drug, you know, if we can, if the cost of these drugs come down sufficiently, the reality is that a higher fraction of our patients will be able to access those drugs and or…if you like a lowered bar for the indication’*

Consultant 4

(2) Evidence for efficacy and safety

There was agreement that evidence for the efficacy and safety of biosimilars was important in the decision to make the switch. Most were happy to accept the licence provision by the regulatory body as sufficient evidence to enable switching.

*‘as long as the other data in terms of efficacy and safety is there, which with biosimilar I believe is there and they have been approved by the FDA (Food and Drug Agency) and the EMA (European Medicines Agency) so they have been regulatory body approved’*

Consultant 2

Alongside efficacy, most of the consultants referenced adverse effects and perceived these to be similar to the biologic.

*“we have not as at least as I am aware and again I have not studied the data in detail but I am not aware that we have had anything that is outside the sort of normal envelope of expected side effects”*

Consultant 4

One consultant did report waiting to introduce biosimilars in their clinical practice until there were more data to underpin the switch.

Manufacturer reputation was another concern raised, with one consultant stating that if they knew the manufacturers, they could trust their quality assurance (QA) processes.

*‘Quality assurance over time will actually be a bigger challenge with vendors that we are not aware of’*

Consultant 5

One concern that was voiced was that because they are less expensive, the adverse event profile is likely to be worse.

*‘so we have now made patients aware that we are using a what do you call it a ‘cheaper compound’ and, and, and so I would expect that we would have a slightly higher likelihood of reporting adverse outcomes, so essentially judging them properly would be a challenge for us’*

Consultant 5

As a consequence of the comparability of the efficacy and adverse effect profiles, most consultants were quite clear that monitoring should remain the same as if the patient had remained on the biologic.

*‘Exactly the same way that we measure normally as well, and we are actually quite generous in the way that we monitor our patients’*

Consultant 5

However, some consultants felt as though they might want to increase the monitoring slightly in the initial stages, at least until the data underpinning each switch were more robust and reliable.

They suggested that this routine monitoring would also assist with determining if the medicine was having an effect and whether they could contemplate stretching dose intervals or reducing doses. This more intense monitoring at the start was echoed by two patients, who felt that it would be wise to monitor more closely in the first few months.

(3) Team roles

In terms of who should be responsible for communicating with patients about the switching process, the consultants felt that this should be the responsibility of the doctor or specialist nurse.

*“well I think you have to be I think a lot of the patients would be wary without you know if they just met say someone they had not met before who said I am going to change you, do not worry your consultant is fine with this I think they’d probably want to hear the consultant say this is the right thing to do as far as I am concerned but in terms of them having cos doctors I do not tend to have a lot of time to and probably are not so good at just having lengthy sort of reassuring discussions so that is something that might be better done by nurses I think”*

Consultant 3

A view was expressed that pharmacists were not the best professionals to have an initial discussion with patients if there was not a pre-existing relationship present that supported a confidence-building conversation. Consultant 5 also thought that the responsibility for this discussion probably resided with the doctor. It was raised that in order to have this discussion, additional resources would probably be needed, and there was uncertainty over whether they had the personnel available to deliver the enhanced patient contact required for the switching programme.

(4) Effective communication of information during switching
  (a) Subtheme was ‘What patients want to know’

There was an identified need to have a process for switching patients from biologics to biosimilars, and this needed to be approached carefully. Patients highlighted the need to have time to process the information and think about questions for the medical team.

*“theoretically then, you could send a letter out in three months’ time we are thinking of doing this if there’s any issue can we perhaps talk with you face to face”*

Etanercept originator FG Pt 6

At the time of this study, the terminology and concept of switching to biosimilar medicines were new and not well understood. Some consultants expressed the view that patients should be given time to review and absorb this information prior to being asked to change the brand from biologic to biosimilar medicine. The option to revert to the originator medicine, as agreed by both the consultant and patient groups, is something that should be offered. This was viewed as particularly important by patients who were generally positive about the switch but wanted options available if they did not react well to the change.

*“if it becomes clear that there is a very obvious change in efficacy of the treatment, are patients able to go back to the original version. If, if that is true, then I think you say to people, look its identical, its fine, we are so confident that it will be identical, if necessary we can take you back to the other treatment if you need to.”*

Infliximab originator FG Pt 1

  (b) Subtheme was ‘How it should be communicated’

Consultants and patients felt as though a mixture of written and face-to-face information should be provided to patients about the switch from biologics and biosimilar medicines. Most of the consultants felt as though there was a significant amount of information to be taken in by patients and that, sometimes, this could be too much. Many patients referred to a simple sheet or series of frequently asked questions (FAQs) to use as a resource to keep the amount of information to a minimum, signposting to other materials if they wanted to obtain further details.

*“Yeah, this would be better if you had a simple sheet, because I often actually do not bother with reading up, it would be nice to have a choice, while producing a large amount of literature can be very expensive, if you do a simple link to a website, could actually keep a reference when you need to”*

Infliximab originator FG Pt 1

Consultants explained that by providing information up front, you will avoid unnecessary anxiety later in their treatment. There was also a suggestion that it is better, and patients have more confidence in information provided by the hospital rather than the manufacturers.

Consultant participants felt that the information provided should contain details about the reasons for switching in a transparent way and that safety and efficacy were comparable. It was also important for patients to know that this is happening in other countries, and there was some mention of using the branded versus generic example to illustrate the points being made. One consultant wanted the cost savings to be made explicit in the information given to patients.

*“I mean I guess it would be helpful to you know, I mean patients need to understand the savings and perhaps give them some examples of what you could do with that if you if this works for you this releases it is a bit like that television programme you know this is 20 hip replacements a year”*

Consultant 3

## 4. Discussion

The purpose of this study was to explore the views and perceptions of patients receiving treatment with biologics and the consultants managing their disease to identify barriers and enablers to switching from a biologic to their equivalent biosimilars. At the time of this study, it is important to note that there were very few patients prescribed biosimilar infliximab or biosimilar etanercept in the United Kingdom. No patients had received a biosimilar prescription at Cambridge University Hospitals NHS Foundation Trust. In 2016, the scarcity of comparative studies limited our ability to validate or challenge our approach [13]. Nonetheless, it provided invaluable initial insights from patients and consultants regarding switching in complex conditions. These early data facilitated the development of local guidelines at Cambridge University Hospitals outlining the information necessary for patients and healthcare professionals considering a transition to biosimilar medications, which would later go on to inform regional and national guidance.

Our results are similar to those reported in a 2023 systematic review, which revealed that patients lacked sufficient knowledge about biosimilars, leading to doubts about their clinical effects and regulatory approval process, potentially affecting their attitudes negatively [13].

While both patients and consultants accepted the financial benefits of switching to biosimilars, it seems that they are less likely to accept this if the money saved just goes into the NHS baseline. Reassurance that any savings will contribute to patient care elsewhere is an important message that will encourage consultants to switch medicines and incentivise patients to accept them [24,25,26,27,28].

There is a drive to switch both biologic-naïve and existing patients onto biosimilar medicine in the National Health Service (NHS). However, the literature does describe the hesitancy in some specialist areas, often when the clinician is unfamiliar with the biosimilars as a concept [17,25]. With some consultants concerned about equal efficacy and others about the quality of production, this needs to be addressed to increase the initiation of switches by consultants.

While clinical trials and real-world data demonstrate the comparative immunogenicity and efficacy of biologics and biosimilar medicines, it is important to highlight that there is no medicine that is 100% safe and that both biologics and biosimilar medicines present associated risks and undesirable effects [29,30,31].

Pharmaceutical companies that manufacture biosimilar medicines follow extensive regulatory processes within the United Kingdom. However, the requirement for clinical trials is designed to demonstrate equivalence to the reference biologic [32]. There was caution from consultant participants surrounding newer companies producing the biosimilar and being less confident with their quality assurance (QA) processes, as well as the outcomes of adverse drug reactions and side effects. It is well documented from more recent studies that physician comfort with originator biologics instead of biosimilars can be a barrier to prescribing biosimilar medicines [33]. They also highlighted that if the regulatory bodies have approved the biosimilar, then there is a reasonable expectation that they will perform in an equally effective manner [6]. These views demonstrate that there was variation in the understanding of biosimilars and their manufacturing process within the consultant group. It is unclear why medical practitioners are concerned about the differences in practices and outputs and whether they know the company or not. With private consulting companies recommending that biologic companies highlight manufacturing processes as differentiators to protect their products, this could potentially explain the origin of some of these concerns [32].

Similar concerns regarding switching from branded medicines to generics, e.g., tablets, have been reported [34]. A questionnaire-based cross-sectional study concluded that a high proportion of consultants believe that generic medicines are of poorer quality, calling the manufacturing process into question, which impacts their reluctance to prescribe [30,35]. Regarding the concern that the medicine being less expensive could suggest the adverse event profile is likely to be worse, it could be due to a lack of awareness of why the cost difference exists, for example, the cost of creating an original treatment compared to that of a biosimilar and the need to recoup these costs plus maximise profit for shareholders [36]. In this study, in both the patient and consultant sessions, a consultant explained they felt the lower the cost, the higher the adverse event profile; however, they could see how a lower cost of treatment could result in better access to patients with milder conditions or for other diseases.

The pharmacist has a pivotal role in supporting other healthcare professionals and patients in pharmacovigilance reporting and adds value as part of the wider multidisciplinary team [30,37,38]. Researchers have shown that patients sought information and gained knowledge about the policy changes either through self-research or from their healthcare providers, such as rheumatologists or pharmacists [39,40].

There were mixed feelings from consultants regarding monitoring post-switching. Some wanted to increase monitoring to reassure their concerns regarding disease activity and safety in line with National Institute of Health and Care Excellence (NICE) guidelines. While others felt it would not be necessary to increase monitoring [21]. Closer monitoring of patients overall provides an opportunity to review the patient and consider if it is appropriate to reduce the dose, extend the interval, or withdraw treatment. Like consultants, patients also required reassurance from the promise of adequate monitoring of switches. Cost-effective approaches to doing this are required.

Professional roles are important; a study conducted prior to ours found that patients did not mind if they were given a biologic or biosimilar, as they intended to trust their doctor’s option [22]. A consultant acknowledged that doctors would unlikely have the time required to have this discussion with each of their patients but favoured nurses as being more acceptable to support due to established patient relationships. In more recent literature, it has been documented that the pharmacy team is best suited to drive the biosimilar adoption and transition process [41]. In this study, it was felt that the pharmacist could play a role both in educating consultants regarding treatment effectiveness and safe manufacturing processes and equally in providing the conduit for patients to report any adverse drug reactions or any concerns related to biosimilar medicine [16].

When this study was conducted, some consultant participants interviewed were not accustomed to collaborating with pharmacists in clinic settings at Cambridge University Hospitals. This was apparent in their responses, as they believed that doctors and nurses were more suited to initiate discussions with patients regarding switching medications. Moreover, they acknowledged potential challenges with staffing resources that could hinder the implementation of the switch programme, presenting an opportunity to integrate a pharmacist within clinics to facilitate the switch programme and assume a leadership role. This proposed approach aligns with findings from other studies [39]. If this study were replicated, it is hypothesised that consultants’ responses would likely vary due to increased experience working with the pharmacy team. For instance, embedding a pharmacist in the gastroenterology inflammatory bowel disease standards and the practice of patient-centred care, which has successfully expanded into rheumatology and dermatology clinics, underscores the importance of pharmacist involvement [19,40,41]. Pharmacists, as medicinal product specialists, can mitigate barriers and dispel misconceptions about for other healthcare professionals and patients, potentially streamlining communication and improving adherence to treatments. This demonstrates the positive impact of having a clinical pharmacist integrated into the clinical team to facilitate the switching process [42].

Advanced communication regarding a pending biologic switch was a high priority for the consultants and patient group, with the consensus reached at 3 months to give them time to consider any questions and then to be offered the opportunity to discuss these questions with a member of the team. They wanted it to sound more like an advisory process than ‘the decision has already been taken.’ Educational and communication interventions have been shown to significantly decrease the rate of brand switchbacks [24,35,43,44,45]. In addition, initiatives targeting both healthcare professionals, patients, and the public about biosimilars, with education, guidelines, and clear recommendations and support from regulatory authorities, would be required to enhance the confidence and appropriate prescribing of clinicians about biologics and biosimilars through academic detailing [46].

The proposed format for disseminating information suggests the creation of a simple information sheet and/or frequently asked questions (FAQs) document, complemented by an electronic link for patients to access at their convenience. Health literacy was the most reported factor affecting patient learning, followed by anxiety and illness conditions. Recognising that patients have diverse learning styles and should be assessed, this approach provides varied preferences in information consumption [47]. Additionally, there was an acknowledgement of the valuable resources provided by manufacturers, such as DVDs and brochures, which could serve as informative tools for patients. Reference to materials from patient support groups was deemed essential for individuals seeking more comprehensive information. However, patients expressed a desire for materials to be more reflective of their experiences and realistic about potential side effects, such as injection site pain. Furthermore, there was a request for real-life examples from patients who had undergone the medication switch, highlighting the importance of incorporating patients’ narratives to enhance understanding and empathy. This comprehensive approach to information provision aims to empower patients with relevant and relatable resources, ultimately fostering informed decision-making and patient satisfaction [48,49,50].

Considering the strengths and limitations of our study, we did not undertake double coding, but co-researchers did re-read the transcripts and thematic analysis. A limitation of the focus groups was that there was not enough time to repeat them; therefore, it is not clear if data saturation was reached. While this study captured representation from gastroenterology, rheumatology, and dermatology, a limitation was that the sample size was too small for the infliximab originator focus group. We aimed for a larger sample for the infliximab originator focus group and received a significant number of willing patient participants. However, recruitment failed due to the date specified being unsuitable for participants. It did provide an opportunity to trial the topic guide and allow the development of facilitation skills prior to the etanercept originator focus group. It was also important to gain contributions from a patient group that receives infusions rather than self-administering, as there are slightly different considerations. Further work should be conducted with consideration for equality, diversity, and inclusive medicine and how we engage with patients from marginalised groups in research who are prescribed biosimilar medicines [51]. Socioeconomic class and ethnic background were not captured in this study but were broadly representative of the population in the area.

## 5. Conclusions

Key study findings are that there is a lack of knowledge or perhaps confidence regarding biosimilars (their efficacy, side effect profile, adverse drug reactions, and immunogenicity) in relation to their biologics from consultants. This impacts the willingness and confidence to prescribe biosimilar medicines, which in turn can affect patients’ perceptions and the success of treatment post-switch.

The patient needs to be assured that the process is not just a cost-saving exercise but will allow additional services to be provided across the NHS to patients, that the treatments are equivalent to their biologics, effective, and equally safe as adverse drug reactions were a concern for the patients, and that an option to revert back would be available to them.

Pharmacists working closely with consultants to ensure education around biosimilar medicine is very important to ensure confidence as well as pharmacovigilance in ensuring details of adverse drug reactions (ADRs) and details of the brands patients are prescribed are recorded to monitor patient outcomes as well as provide leadership for the switch programme.

## Figures and Tables

**Table 1:** Demographics and diagnosis of patient participants.

	Infliximab Originator Focus Group	Etanercept Originator Focus Group
**Number of patients**	2	7
**Females**	1	5
**Age**	34–55	28–79
**Diagnosis**		
**Spondyloarthritis**		2
**Rheumatoid arthritis and psoriasis**		1
**Rheumatoid arthritis**	1	4
**Crohn’s disease**	1	

## Data Availability

The research data are not publicly available.

## Appendix A. Topic Guides/Questions for Patient Focus Groups

**Table A1:** Topic guides/questions for patient focus groups.

A presentation explaining the difference between biologic medicines and biosimilar medicines was delivered prior to asking questions.	
**Questions**	**Prompts**
What are your initial thoughts on the content of the presentation?	Concerns? Safety? Cost? Treatment for other patients?
Will switching to a biosimilar affect anything about how you take your medication in any way?	Frequency? Attendance at appointments?
If we change your medication to a biosimilar product, how should we introduce the switch?	Via letter, face-to-face consultation? Who should switch your medication? Doctor, Nurse, Pharmacist? What should your medication be switched?
If we change to the biosimilar, what information should be given to patients?	Who do you see is the correct person or method of receiving this information? Patient information leaflets? Letter in the post? Social media, i.e., Apps (applications)? What information can we provide to encourage patients to adopt the change?
Is it necessary to be monitored if prescribed a biosimilar, and if so, how should this be done?	How should this be done? Via telephone, face to face? How often? How soon after the first dose? Who should monitor you? What should be monitored? Drug levels, disease activity?
Thank you for your input and time. Is there any advice you could give us?	

## Appendix B. Topic Guides/Questions for Consultant Interviews

**Table A2:** Topic guides/questions for consultant interviews.

Questions	Prompts
What are your views about switching patients from one biosimilar to another biosimilar?	Cost? Experience? Patient registers?
What are the important drivers for switching?	
What are the roles of the different members of the team?	Doctor, specialist nurse, pharmacist
What should be monitored once the patient has been switched to a biosimilar?	What should be monitored? Drug levels, disease activity?
What information do you think the patient should be given?	In what form? i.e., patient information leaflets?
How should the information be delivered to the patient?	Content? Letter in the post? Social Media, i.e., applications? Presentations?
How do you see the future of biosimilars?	Pathways? Cost changes every year?
